# Control of mRNA Splicing by Intragenic RNA Activators of Stress Signaling: Potential Implications for Human Disease

**DOI:** 10.3389/fgene.2019.00464

**Published:** 2019-05-14

**Authors:** Raymond Kaempfer, Lena Ilan, Smadar Cohen-Chalamish, Orli Turgeman, Lise Sarah Namer, Farhat Osman

**Affiliations:** Department of Biochemistry and Molecular Biology, The Institute for Medical Research Israel-Canada, The Hebrew University-Hadassah Medical School, Jerusalem, Israel

**Keywords:** mRNA splicing control, intragenic RNA activators of PKR, activation of PKR, eIF2α phosphorylation, PKR silencer elements, *TNF-α* gene, *β-globin* gene, human β-thalassemia mutations

## Abstract

A critical step in the cellular stress response is transient activation of the RNA-dependent protein kinase PKR by double-helical RNA, resulting in down-regulation of protein synthesis through phosphorylation of the α chain of translation initiation factor eIF2, a major PKR substrate. However, intragenic elements of 100–200 nucleotides in length within primary transcripts of cellular genes, exemplified by the *tumor necrosis factor (TNF)-α* gene and fetal and adult *globin* genes, are capable of forming RNA structures that potently activate PKR and thereby strongly enhance mRNA splicing efficiency. By inducing nuclear eIF2α phosphorylation, these PKR activator elements enable highly efficient early spliceosome assembly yet do not impair translation of the mature spliced mRNA. The *TNF-α* RNA activator of PKR folds into a compact pseudoknot that is highly conserved within the phylogeny. Upon excision of *β-globin* first intron, the RNA activator of PKR, located in exon 1, is silenced through strand displacement by a short sequence within exon 2, restricting thereby the ability to activate PKR to the splicing process without impeding subsequent synthesis of β-globin essential for survival. This activator/silencer mechanism likewise controls splicing of *α-globin* pre-mRNA, but the exonic locations of PKR activator and silencer sequences are reversed, demonstrating evolutionary flexibility. Impaired splicing efficiency may underlie numerous human β-thalassemia mutations that map to the *β-globin* RNA activator of PKR or its silencer. Even where such mutations change the encoded amino acid sequence during subsequent translation, they carry the potential of first impairing PKR-dependent mRNA splicing or shutoff of PKR activation needed for optimal translation.

## Introduction

Phosphorylation of the α-chain of eukaryotic translation initiation factor 2 (eIF2α) is critical for mounting the integrated cellular stress response (Harding et al., 2003; Muaddi et al., 2010). Transient phosphorylation of eIF2α blocks GDP/GTP exchange needed for recycling of eIF2 between rounds of protein synthesis, inducing translational repression (Sonenberg and Hinnebusch, 2009). The RNA-dependent protein kinase PKR is a prominent eIF2α kinase having a major role in the IFN-mediated antiviral response. IFNs, including IFN-γ, induce high levels of *PKR* gene transcription in the cell (Stark et al., 1998). To become activated, PKR must undergo ATP-dependent *trans*-autophosphorylation upon engaging, through its tandem RNA binding motifs, double-stranded RNA generated during virus replication (Meurs et al., 1990). Highly ordered double-stranded RNA structures rather than specific sequences are needed to activate PKR (Bevilacqua and Cech, 1996). Once activated by double-stranded RNA, PKR will phosphorylate eIF2α, blocking translation and virus spread from infected cells (Stark et al., 1998).

We review here the discovery and mode of action of a novel class of regulatory RNA elements inside cellular genes that activate PKR to control thereby not only their translation but in particular, enhance their mRNA splicing. Once transcribed into single-stranded RNA, these short non-coding elements fold into structures that act in *cis* to potently activate PKR, rendering splicing highly efficient (Osman et al., 1999; Ilan et al., 2017; Namer et al., 2017) or repressing translation of the encoded mRNA (Ben-Asouli et al., 2002; Cohen-Chalamish et al., 2009), in each case by inducing eIF2α phosphorylation. We address potential implications of these RNA elements for human disease.

## Regulation of Gene Expression by Intragenic Elements That Activate PKR

Linear double-stranded RNA, generated in the course of virus infection, was considered to be the classical activator of PKR. That notion was shattered by the discovery of short elements within cellular genes that once transcribed, fold into RNA structures capable of activating PKR even more effectively and use this property to control gene expression. Thus, human *IFN-γ* mRNA contains a 5′-terminal 203-nt element that folds into a pseudoknot that potently activates PKR, inducing thereby eIF2α phosphorylation and attenuating its own translation by an order of magnitude (Ben-Asouli et al., 2002; Cohen-Chalamish et al., 2009). This negative feedback loop prevents induction of pathological hyper-inflammation by limiting production of IFN-γ, a prominent inflammatory cytokine (Ben-Asouli et al., 2002). This intragenic element also couples *IFN-γ* mRNA translation to the level of PKR in the cell (Ben-Asouli et al., 2002). Extensive mutational analysis combined with structure probing showed that the RNA activator of PKR is denatured by ribosome passage and undergoes dynamic refolding to allow PKR activation in the course of translation (Cohen-Chalamish et al., 2009). Because both activation of PKR and phosphorylation of eIF2α substrate are transient events, followed promptly by dephosphorylation that inactivates PKR while restoring eIF2α activity, intragenic RNA activators of PKR function locally as *cis*-acting control elements (Osman et al., 1999; Ben-Asouli et al., 2002; Namer et al., 2017).

## *TNF-α* mRNA Splicing Depends on Activation of PKR and Phosphorylation of Its EIF2α Substrate

**FIGURE 1:**
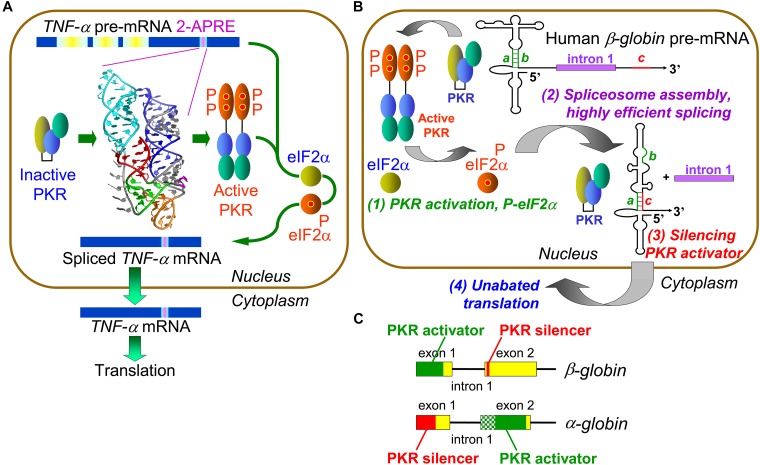
Control of mRNA splicing by intragenic RNA activators of PKR and eIF2α phosphorylation. **(A)** An RNA element in *TNF-α* pre-mRNA controls splicing by activating PKR and thereby inducing eIF2α phosphorylation. The 104-nt *TNF-α* activator of PKR (2-APRE), located within the 3′-UTR, potently activates PKR through its pseudoknot structure that constrains the RNA into two parallel helices, each capable of binding a PKR monomer, facilitating PKR dimerization on RNA needed for kinase activation. Once PKR is activated, it must induce eIF-2α phosphorylation to enable efficient splicing of *TNF-α* mRNA. **(B)** Regulation of *β-globin* pre-mRNA splicing by an intragenic RNA activator of PKR and silencing of the PKR activator upon intron excision. (1) Within the RNA activator of PKR, consisting of 5′-terminal 124 nt within exon 1, 5 bp helix *a–b* (green) is critical for activity. Once PKR is activated, it must induce eIF-2α phosphorylation to enable efficient spliceosome assembly and mRNA splicing (2). Upon excision of intron 1, sequence *c* located near the start of exon 2 (red) displaces strand *b* from strand *a*, a rearrangement that silences the ability of mature *β-globin* mRNA to activate PKR (3). This renders activation of PKR transient, serving solely to promote splicing yet allowing for unimpeded synthesis of β-globin protein (4) (after Kaempfer et al., 2018). **(C)** Elements that activate PKR or silence PKR activation map into opposite exons in *α-* and *β-globin* pre-mRNA. The core of the *α-globin* RNA activator of PKR is shown in solid green within exon 2; maximal PKR activation also requires upstream RNA sequence shown in shaded green.

The inflammatory cytokine TNF-α is not only critical for protective immunity and the anti-tumor response but also a major mediator of inflammatory diseases. TNF-α is expressed promptly during the immune response, *TNF-α* mRNA levels becoming maximal within 3 h in stimulated human PBMC (Jarrous et al., 1996). To achieve such efficient expression, splicing of *TNF-α* mRNA uses activation of PKR. The adenine analog 2-aminopurine, a competitive inhibitor of ATP in binding kinases, especially PKR, blocks splicing of all three *TNF-α* introns (Jarrous et al., 1996). Splicing of *TNF-α* pre-mRNA is controlled by the 104-nt 2-APRE located within the 3′-UTR (Figure 1A; Osman et al., 1999). This *cis*-acting RNA element activates PKR more potently than does double-stranded RNA and enhances *TNF-α* mRNA splicing by over an order of magnitude when PKR expression is increased (Osman et al., 1999). Mutational analysis, including compensatory mutations that restore base pairing and secondary structure of RNA, showed that the 2-APRE renders nuclear splicing of *TNF-α* pre-mRNA not only strictly dependent on PKR activation but also highly efficient, yet does not cause translational repression (Osman et al., 1999; Namer et al., 2017). In contrast to *TNF-α*, the closely related *TNF-β* (lymphotoxin) gene does not harbor an intragenic activator of PKR and its mRNA is spliced sluggishly; yet, upon transposition of the *TNF-α* element into the *TNF-β* 3′-UTR, splicing of *TNF-β* pre-mRNA became as efficient as that of *TNF-α*, showing that the 2-APRE functions as an autonomous splicing control element (Osman et al., 1999; Namer et al., 2017).

Protein kinase RNA-activated activation requires its homodimerization on the activating RNA to permit *trans*-autophosphorylation leading to kinase activation (Zhang et al., 2001; Dey et al., 2005). Given that activation of PKR requires at least 33 and optimally 80 base pairs of double-helical RNA (Manche et al., 1992; Bevilacqua and Cech, 1996), how could the 2-APRE, having only 104 nt, activate PKR so potently? Extensive genetic analysis, validated by gain-of-function mutations, revealed that the *TNF-α* RNA activator of PKR folds into a compact pseudoknot that constrains the RNA into two double-helical stacks with parallel axes (Figure 1A), each long enough to bind a PKR monomer, promoting efficient kinase dimerization enabling activation (Namer et al., 2017). The *TNF-α* pseudoknot is highly conserved in the phylogeny over 400 million years, from teleost fish to humans. Indeed, turbot 2-APRE RNA activates human PKR and enhances human *TNF-β* mRNA splicing as effectively as does the human element (Namer et al., 2017).

Local activation of PKR not only enhances *TNF-α* mRNA splicing but also increases protein yield correspondingly, without repressing translation (Namer et al., 2017). Surprisingly, we discovered that PKR activation promotes efficient *TNF-α* mRNA splicing by inducing eIF2α phosphorylation (Figure 1A). Expression of non-phosphorylatable mutant eIF2α abrogated PKR-dependent splicing. Phosphorylation of eIF2α is not only strictly needed but also sufficient to achieve highly efficient splicing. Blocking rapid dephosphorylation of eIF2α with salubrinal, which increases phospho-eIF2α globally in the cell (Boyce et al., 2005), sufficed to raise splicing the efficiency of *TNF-β* pre-mRNA to that of *TNF-α* pre-mRNA (Namer et al., 2017). eIF2α phosphorylation upregulates *TNF-α* mRNA splicing in human PBMC, demonstrating its physiological relevance. Therefore, stress-induced PKR-mediated eIF2α phosphorylation has not only a major role in down-regulating translation but also plays a key positive role in rendering splicing highly efficient.

## Intragenic RNA Activators of PKR Control *Globin* Gene Expression at mRNA Splicing

To analyze the molecular mechanism underlying highly efficient splicing of *TNF-α* mRNA induced by its intragenic RNA activator of PKR and mediated by eIF2α phosphorylation, we offered *in vitro* transcribed *TNF-α* precursor RNA as substrate for splicing in HeLa cell nuclear extract. That attempt failed, owing to prompt and complete degradation of *TNF-α* pre-mRNA. However, it led to our discovery that splicing of *β-globin* exon1-intron1-exon2 template, serving as positive control for splicing (Krainer et al., 1984), also depends strictly on the activation of PKR (Ilan et al., 2017). This came as a surprise, given that *globin* gene expression has long served as a paradigm for eukaryotic gene regulation. Indeed, splicing of human *α-globin*, *β-globin* as well as fetal *γ-globin* pre-mRNAs depends heavily on PKR activation induced by intragenic RNA activator elements (Ilan et al., 2017). Hence, PKR activation is used more broadly within the human genome beyond inflammatory cytokine genes, to control mRNA splicing. Excision of *β-globin* intron 1, the first splicing event, was blocked by anti-PKR antibodies as well as by PKR depletion, where it could be restored with recombinant PKR.

The *β-globin* RNA activator of PKR maps into the first exon (Figure 1B, step 1); mutation of short helix *a–b* in the *β-globin* activator severely impairs both PKR activation and mRNA splicing. Efficient splicing of each of *α-*, *β-* and *γ-globin* pre-mRNA species depends strictly on activation of PKR and nuclear eIF2α phosphorylation and is inhibited by non-phosphorylatable mutant eIF2α or anti-phospho-eIF2α antibodies (Ilan et al., 2017). Activation of PKR and eIF2α phosphorylation are required at an early step in *β-globin* spliceosome assembly, formation of Complex A (Figure 1B, step 2). As shown for *β-globin* pre-mRNA, activation of PKR and phosphorylation of eIF2α mediate splicing not only *in vitro* but also in intact cells (Ilan et al., 2017).

## Intragenic RNA-Mediated Silencing of PKR Activators Upon Splicing

The RNA activator of PKR is contained within *β-globin* exon 1 and thus maintained in spliced mRNA, where it could strongly down-regulate translation as shown for *IFN-γ* mRNA (Ben-Asouli et al., 2002; Cohen-Chalamish et al., 2009), creating a paradox. Yet, during erythroid development, *globin* mRNA is translated massively, reaching 95% of total protein in reticulocytes as compared to <0.1% in proerythroblasts (Nienhuis and Benz, 1977). How is maximal translation achieved? Excision of *β-globin* first intron juxtaposes short 5-nucleotide sequence *c*, located near the start of exon 2, to exon 1, inducing strand displacement within exon 1 that destroys helix *a–b* at the core of the PKR activator, resulting in silencing of the activator once *β-globin* mRNA is spliced (Figure 1B, step 3 and Figure 1C). Mutation of either strand *a* or *c* abrogated silencing whereas compensatory mutation restored it (Ilan et al., 2017). Splicing of *α-globin* pre-mRNA is regulated similarly except that locations of PKR activator and silencer are reversed between exons 2 and 1, demonstrating evolutionary flexibility in control of PKR activation during and upon splicing (Figure 1C). The silencing mechanism allows for highly efficient PKR-dependent splicing, followed promptly by shutoff of PKR activation, to permit undisturbed, maximal translation of the spliced mRNA product (Ilan et al., 2017). This mechanism assures that the ability to activate PKR remains transient, serving only to enable efficient splicing, without hindering globin synthesis (Figure 1B, step 4).

## Intragenic RNA Elements That Activate PKR or Silence PKR Activators are Potential Sources of Human Disease

**FIGURE 2:**
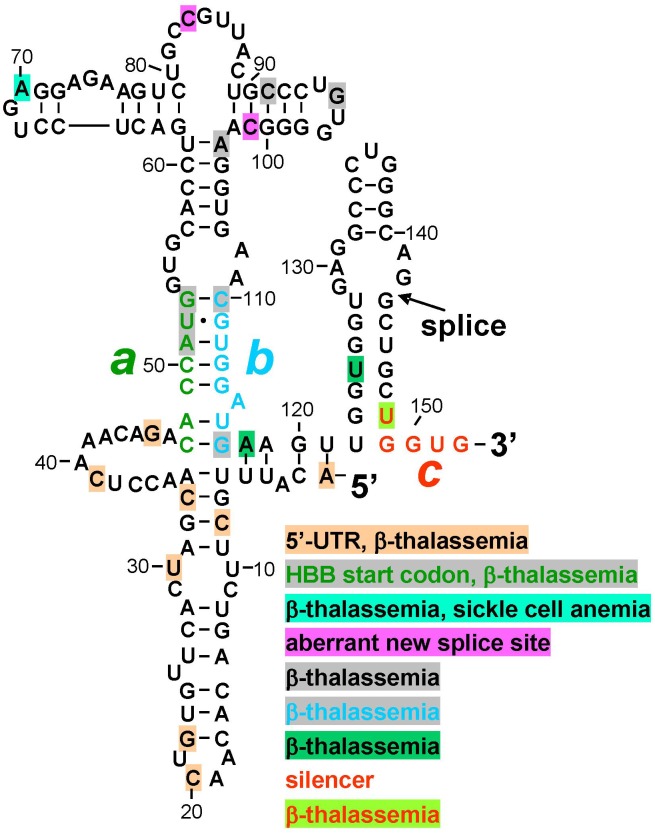
Mutations in human *β-globin* RNA activator of PKR and silencer of PKR activation are associated with β-thalassemia. Structure of the RNA activator of PKR (nucleotides 1–124), determined by in-line probing and mutagenesis, with a key role for helix strands *a* (green) and *b* (cyan). Strand *a* includes the AUG start codon. Position of first splice junction is shown, as is start of exon 2 containing PKR silencer *c* (red), upon excision of intron 1 but before displacement of strand *b* by sequence *c* validated by in-line probing and mutagenesis (see Figure 1B). Nucleotide mutations associated with β-thalassemia in the human gene mutation database (http://www.hgmd.org), are marked by shading in various colors, see text. HBB, human *β-globin* gene.

Protein kinase RNA-activated activator and silencer RNA structures were defined for the human *β-globin* gene (HBB) by truncation, mutational analysis, and in-line probing of the RNA (Figure 1B; Ilan et al., 2017). Figure 2 depicts the resulting RNA secondary structure. The activator of PKR is comprised of nucleotides 1–124 from the 5′ end; truncation of only a few nucleotides from either side sufficed to abrogate PKR activation. The AUG start codon, located at positions 51–53, forms part of strand *a* that generates the helix at the core of the PKR activator. Replacement of CACCA in strand *a*, including A of the start codon, by complementary nucleotides had a pronounced negative effect on splicing efficiency. Replacement of CGUGG in opposite strand *b*, which includes the sequence that engages the AUG codon, by complementary nucleotides largely abrogated splicing efficiency both in cells and *in vitro*, whereas efficient splicing was restored by *ab* double mutation (Ilan et al., 2017).

Inspection of the human gene mutation data base (HBB^1^) shows that numerous human β-thalassemia mutations map to the *β-globin* RNA activator of PKR or to its silencer (Figure 2). Thus, regulatory mutations were reported within the 5′-UTR, many without mechanism. However, C33G mutation reduced the *β-globin* transcript level (Ho et al., 1996) while A1G mutation led, in mouse β-major *globin*, to over twofold decreased mRNA expression (Myers et al., 1986); splicing was not analyzed. Other β-thalassemia mutations map to the AUG initiation codon within strand *a*, to the bifurcation loop structure, to nucleotides in strand *b* that form G-C base pairs with strand *a* to generate the helix indispensable for PKR activation, as well as to nucleotides adjoining strand *b* and the 3′ end of the PKR activator structure (Figure 2). Within silencer sequence *c*, U148A mutation destabilizes base pairing with strand *a*. Because splicing precedes translation, the effect of these mutations may be manifested at the level of PKR activation needed for high splicing efficiency, or at shutoff of PKR activation directly upon splicing, even before they can affect the sequence of the translated β-globin protein product that is more readily diagnosed in patients and traditionally reported on the data base. Thus, although mutation of the AUG start codon can severely impact translation, this codon also has a dual function in controlling splicing, being located at the heart of the RNA activator of PKR and base pairing with the silencer.

Splicing-defective mutations reported within the *β-globin* PKR activator domain (Figure 2) create aberrant splice donor sites that alter protein sequence; aberrant splice donor site mutations are lacking for downstream *β-globin* exons 2 and 3.

Minimal sequences encoding the *α-globin* RNA activator of PKR and silencer (Figure 1C) were defined thus far only by truncation analysis (Ilan et al., 2017); their RNA structure remains to be determined. Therefore, it is too early to perform a similar analysis for *α-globin*. Nonetheless, numerous mutations characterized as leading to α-thalassemia, hemolytic anemia, or variant α-globin proteins (HBA1, HBA2; see footnote 1) map to the PKR activator and silencer domains as delineated at present.

Following the pattern for adult *β-globin*, the RNA activator of PKR of *γ-globin*, the fetal form of *β-globin*, is located within the first exon and *γ-globin* mRNA splicing is strictly dependent on PKR activation and eIF2α phosphorylation (Ilan et al., 2017), but the structure of the *γ-globin* PKR activator element was not yet analyzed.

Mutational analysis of the *TNF-α* RNA activator of PKR (2-APRE, Figure 1A) demonstrated its exquisite sensitivity to mutations, even to a single nucleotide change or base pair inversion, in activating PKR and rendering splicing highly efficient (Namer et al., 2017). Length and nature of the *TNF-α* 3′-UTR impart great lability to the primary transcript, rendering detection and analysis of splicing defects in human patients difficult. Assay of *TNF-α* splicing efficiency necessarily must be done in primary PBMC or transfected cells, using real-time polymerase chain reaction or ribonuclease protection analysis (Namer et al., 2017). It is thus no surprise that among mutations reported as yielding a *TNF-α* phenotype, the human gene mutation data base lacks thus far mutations mapping into the 2-APRE, remote from the coding region. Focus has been on control of *TNF-α* mRNA translation by microRNAs (TNFA; see footnote 1).

## Future Perspectives

The discovery of intragenic elements that once transcribed, control splicing by activating PKR in the nucleus or by silencing the ability to activate PKR, adds a new dimension to the analysis and interpretation of human gene mutations. As shown for the RNA activators of PKR within *IFN-γ* mRNA and *TNF-α* pre-mRNA, even single-nucleotide substitutions or the inversion of a single base pair can lead to loss of the ability of the RNA element to activate PKR (Cohen-Chalamish et al., 2009; Namer et al., 2017). This demonstrates the exquisite sensitivity of the PKR protein molecule to the RNA structure that it must interact with in order to achieve kinase activation, resulting in eIF2α phosphorylation that in turn enhances mRNA splicing efficiency. As shown for *β-globin* pre-mRNA, on the other hand, silencing of the ability of its RNA activator of PKR to activate the kinase, through effective base pairing with RNA encoded by a separate silencer element that induces conformational changes within the RNA activator structure, in this case through strand displacement, is likewise sensitive to mutation (Ilan et al., 2017).

Thus, short intragenic RNA elements that activate PKR or that silence PKR activators are not only essential for controlling efficient mRNA splicing but also create potential etiology for human disease. In a broader sense, this novel perspective may account for and/or contribute to the phenotype of gene mutations analyzed hitherto primarily for their effect on protein sequence. Even where such mutations change the encoded amino acid sequence during subsequent translation in the cytoplasm, they also carry the potential of first impairing PKR-dependent mRNA splicing in the nucleus or the shutoff of PKR activation needed for optimal translation. That concept extends to silent mutations and to mutations that alter amino acid sequence without having a major effect on protein function.

## Author Contributions

OT searched the human gene mutation database. OT and RK analyzed the human mutation data. LI, SC-C, LN, FO, and RK designed and performed the experiments and analyzed the results. RK wrote the manuscript. All authors read and approved the final version of the manuscript for submission.

## Conflict of Interest Statement

The authors declare that the research was conducted in the absence of any commercial or financial relationships that could be construed as a potential conflict of interest.

## Footnotes

1 http://www.hgmd.org

## ABBREVIATIONS

2-APRE: 2-aminopurine response element
PKR: protein kinase RNA-activated
eIF2α: eukaryotic initiation factor 2 α-chain
TNF: tumor necrosis factor
IFN: interferon
nt: nucleotide
PBMC: peripheral blood mononuclear cells
UTR: untranslated region

## References

[B1] Ben-Asouli Y. Banai Y. Pel-Or Y. Shir A. Kaempfer R. (2002). Human interferon-γ mRNA autoregulates its translation through a pseudoknot that activates the interferon-inducible protein kinase PKR. *Cell* 108 221–232. 10.1016/s0092-8674(02)00616-5 11832212

[B2] Bevilacqua P. C. Cech T. R. (1996). Minor-groove recognition of double-stranded RNA by the double-stranded RNA-binding domain from the RNA-activated protein kinase PKR. *Biochemistry* 35 9983–9994. 10.1021/bi9607259 8756460

[B3] Boyce M. Bryant K. F. Jousse C. Long K. Harding H. P. Scheuner D. (2005). A selective inhibitor of eIF2α dephosphorylation protects cells from ER stress. *Science* 307 935–939. 10.1126/science.1101902 15705855

[B4] Cohen-Chalamish S. Hasson A. Weinberg D. Namer L. S. Banai Y. Osman F. (2009). Dynamic refolding of IFN-γ mRNA enables it to function as PKR activator and translation template. *Nat. Chem. Biol.* 5 896–903. 10.1038/nchembio.234 19801993

[B5] Dey M. Cao C. Dar A. C. Tamura T. Ozato K. Sicheri F. (2005). Mechanistic link between PKR dimerization, autophosphorylation, and eIF2α substrate recognition. *Cell* 122 901–913. 10.1016/j.cell.2005.06.041 16179259

[B6] Harding H. P. Zhang Y. Zeng H. Novoa I. Lu P. D. Calfon M. (2003). An integrated stress response regulates amino acid metabolism and resistance to oxidative stress. *Mol. Cell* 11 619–633. 10.1016/s1097-2765(03)00105-9 12667446

[B7] Ho P. J. Rochette J. Fisher C. A. Wonke B. Jarvis M. K. Yardumian A. (1996). Moderate reduction of beta-globin gene transcript by a novel mutation in the 5′ untranslated region: a study of its interaction with other genotypes in two families. *Blood* 87 1170–1178. 8562944

[B8] Ilan L. Osman F. Namer L. S. Eliahu E. Cohen-Chalamish S. Ben-Asouli Y. (2017). PKR activation and eIF2α phosphorylation mediate human globin mRNA splicing at spliceosome assembly. *Cell Res.* 27 688–704. 10.1038/cr.2017.39 28374749 PMC5520854

[B9] Jarrous N. Osman F. Kaempfer R. (1996). 2-Aminopurine selectively inhibits splicing of tumor necrosis factor alpha mRNA. *Mol. Cell. Biol.* 16 2814–2822. 10.1128/mcb.16.6.2814 8649390 PMC231273

[B10] Kaempfer R. Namer L. S. Osman F. Ilan L. (2018). Control of mRNA splicing by noncoding intragenic RNA elements that evoke a cellular stress response. *Int. J. Biochem. Cell. Biol.* 105 20–23. 10.1016/j.biocel.2018.09.021 30282053

[B11] Krainer A. R. Maniatis T. Ruskin B. Green M. R. (1984). Normal and mutant human β-globin pre-mRNAs are faithfully and efficiently spliced in vitro. *Cell* 36 993–1005. 10.1016/0092-8674(84)90049-7 6323033

[B12] Manche L. Green S. R. Schmedt C. Mathews M. B. (1992). Interactions between double-stranded RNA regulators and the protein kinase DAI. *Mol. Cell. Biol.* 12 5238–5248. 10.1128/mcb.12.11.5238 1357546 PMC360457

[B13] Meurs E. Chong K. Galabru J. Thomas N. S. Kerr I. M. Williams B. R. (1990). Molecular cloning and characterization of the human double-stranded RNA-activated protein kinase induced by interferon. *Cell* 62 379–390. 10.1016/0092-8674(90)90374-n 1695551

[B14] Muaddi H. Majumder M. Peidis P. Papadakis A. I. Holcik M. Scheuner D. (2010). Phosphorylation of eIF2α at serine 51 is an important determinant of cell survival and adaptation to glucose deficiency. *Mol. Biol. Cell* 21 3220–3231. 10.1091/mbc.E10-01-0023 20660158 PMC2938387

[B15] Myers R. M. Tilly K. Maniatis T. (1986). Fine structure genetic analysis of a beta-globin promoter. *Science* 232 613–618. 10.1126/science.3457470 3457470

[B16] Namer L. S. Osman F. Banai Y. Masquida B. Jung R. Kaempfer R. (2017). An ancient pseudoknot in TNF-α pre-mRNA activates PKR, inducing eIF2α phosphorylation that potently enhances splicing. *Cell Rep.* 20 188–200. 10.1016/j.celrep.2017.06.035 28683312

[B17] Nienhuis A. W. Benz E. J. (1977). Regulation of hemoglobin synthesis during the development of the red cell. *N. Engl. J. Med.* 297 1318–1328. 10.1056/NEJM197712152972404 335250

[B18] Osman F. Jarrous N. Ben-Asouli Y. Kaempfer R. (1999). A cis-acting element in the 3′-untranslated region of human TNF-α mRNA renders splicing dependent on the activation of protein kinase PKR. *Genes Dev.* 13 3280–3293. 10.1101/gad.13.24.3280 10617576 PMC317206

[B19] Sonenberg N. Hinnebusch A. G. (2009). Regulation of translation initiation in eukaryotes: mechanisms and biological targets. *Cell* 136 731–745. 10.1016/j.cell.2009.01.042 19239892 PMC3610329

[B20] Stark G. R. Kerr I. M. Williams B. R. Silverman R. H. Schreiber R. D. (1998). How cells respond to interferons. *Annu. Rev. Biochem.* 67 227–264. 10.1146/annurev.biochem.67.1.227 9759489

[B21] Zhang F. Romano P. R. Nagamura-Inoue T. Tian B. Dever T. E. Mathews M. B. (2001). Binding of double-stranded RNA to protein kinase PKR is required for dimerization and promotes critical autophosphorylation events in the activation loop. *J. Biol. Chem.* 276 24946–24958. 10.1074/jbc.M102108200 11337501

